# Supplementary material for: Impact of herpes zoster vaccination on incident dementia: A retrospective study in two patient cohorts
Source: PLoS One. 2021 Nov 17;16(11):e0257405. doi: 10.1371/journal.pone.0257405 (PMC8597989; doi:10.1371/journal.pone.0257405)
Supplement: S1 Appendix — (DOCX) [file pone.0257405.s001.docx]

**Supplementary Appendix**

**Table of Contents**

**Variable definitions** ……………………………………………………………………. pages 2 - 6

**Propensity Score (PS) and Inverse Probability of Treatment Weighting (IPTW**)…. page 7

**Distribution of herpes zoster (HZ) infection and antiviral therapy**…………………. page 8

**Balanced covariates following PS and IPTW**………………………………………..... pages 9-10

**Expanded Competing Risk Hazard Models VHA data**…….……………………...…. page 11

**Expanded Cox Proportional Hazard Models MarketScan data**………….…………. page 12

**Secondary outcome – Alzheimer’s Disease**……………………..………….…………. pages 13-14

**Eligibility sampling diagrams**…………………………………………………………..pages 15-16

**References**……………………………………………………………………………….. page 17

| **Table S.1. Variable definitions** | |
| --- | --- |
| **Variable** | **Definition** |
| **Outcome and Exclusions** | |
| Dementia Exclusion Codes (used in washout) | \| **ICD-9 – Codes** \| \| \| \| \| \| \| \| \| --- \| --- \| --- \| --- \| --- \| --- \| --- \| --- \| \| 046.1x \| 046.3 \| \| \| 290.x \| \| 291.1 \| 291.2 \| \| 294.0 \| 294.1x \| \| \| 294.2x \| \| 331.x \| 332.x \| \| 333.0 \| 333.4 \| \| \| 438.0 \| \| 780.93 \| 797 \| \| **ICD-10 – Codes** \| \| \| \| \| \| \| \| \| A81.0x \| \| A81.2 \| F01.x \| \| F02.x \| \| F03.x \| \| F04 \| \| F05 \| F10.26 \| \| F10.27 \| \| F10.96 \| \| F10.97 \| \| G10 \| G20 \| \| G21.1x \| \| G21.2 \| \| G21.3 \| \| G21.4 \| G21.8 \| \| G21.9 \| \| G23.x \| \| G30.x \| \| G31.0x \| G31.1 \| \| G31.2 \| \| G31.83 \| \| G31.84 \| \| G31.85 \| G31.89 \| \| G31.9 \| \| G91.x \| \| G93.7 \| \| G94 \| I69.91 \| \| R41.2 \| \| R41.3 \| \| R41.81 \| \|  \|  \| \|  \| \|  \| |
| Dementia Main Outcome Codes | \| **ICD-9 – Codes** \| \| \| \| \| \| \| \| \| \| \| \| --- \| --- \| --- \| --- \| --- \| --- \| --- \| --- \| --- \| --- \| --- \| \| 290.0 \| \| 290.1x \| \| 290.2x \| \| 290.3 \| \| 290.4x \| \| 294.1x \| \| 294.2x \| \| 331.0 \| \| 331.1x \| \| 331.2 \| \|  \| \|  \| \| **ICD-10 – Codes** \| \| \| \| \| \| \| \| \| \| \| F01.x \| F02.x \| \| F0.x \| \| G3.x \| \| G31.0x \| \| G31.1 \|   - 2 code occurrences on different days within the same 12-month period  - Date of dementia onset is the first date of diagnosis when criteria met |
| Alzheimer’s Disease – secondary outcome | **ICD-9 – Codes**: 331.0;  **ICD-10 – Codes:** G30.x  - 2 code occurrences on different days within the same 12-month period  - Date of Alzheimer’s onset is the first date of diagnosis when criteria met |
| Dementia medications | Donepezil, Rivastigmine, Galantamine, Memantine |
| **Covariates – measured on or before index date. These variables are used in PS models.** | |
| Type II Diabetes | ICD-9 code: 250.x0, 250.x2, 357.2, 362.0x, 366.41;  ICD-10 code: E11.x, E08.42, E09.42, E13.42, E08.36, E09.36, E13.36  - 2 code occurrences in any 24-month period on/prior to index |
| Smoking/nicotine dependence | ICD-9 code: V15.82, 305.1;  ICD-10 code: Z87.891, Z72.0, F17.20x, F17.21x  CPT code: 99406, 99407  HCPCS code: G0436, G0437;  Present in health factor data as current smoker (VHA only)  - Single occurrence on/prior to index date |
| Obesity | ICD-9 code: 278.00, 278.01;  ICD-10 code: E66.9, E66.01;  CPT code: 99401, 99402;  HCPCS code: G0446, G0447, G0473  Last BMI on/before index is ≥ 30 (VHA only)  - Single occurrence on/prior to index date or last BMI before index is ≥ 30 |
| Hypertension | ICD-9 code: 401.x;  ICD-10 code: I10  - Single occurrence on/prior to index date |
| Hyperlipidemia | ICD-9 code: 272.0, 272.1, 272.2, 272.4;  ICD-10 code: E78.0-E78.2, E78.4-E78.5  - Single occurrence on/prior to index date |
| Stroke or cerebrovascular accident | ICD-9 code: 431.x, 434.x, 438.x;  ICD-10 code: I61.x, I63.3x, I63.4x, I63.5x, I63.6, I63.8, I63.9, I66.x, I69.1x, I69.2x, I69.3x, I69.8x, I69.9x  - Single occurrence on/prior to index |
| Ischemic heart disease | ICD-9 code: 410.x – 414.x;  ICD-10 code: I20.x – I25.x  - Single occurrence on/prior to index |
| Congestive heart failure | ICD-9 code: 398.91, 402.11, 402.91, 404.11, 404.13, 404.91, 404.93, 428.x;  ICD-10 code: I09.81, I11.0, I13.0, I13.2, I50.x  -Single occurrence on/prior to index |
| Atrial fibrillation of flutter | ICD-9 code: 427.3x;  ICD-10 code: I48.x  - Single occurrence on/prior to index |
| Asthma | ICD-9 code: 493.x;  ICD-10 code: J45.x  - Single occurrence on/prior to index |
| COPD | ICD-9 code: 491.2x, 496;  ICD-10 code: J44.x  - Single occurrence on/prior to index |
| Traumatic brain injury | ICD-9 code: 800.x-804.x, 850.x-854.x, 905.0, 907.0, 959.01, V15.52;  ICD-10 code: S01.90x, S02.x, S04.x, S06.x, S07.x, S09.8x, S09.9x, Z87.820  - Single occurrence on/prior to index date |
| Vitamin B12 deficiency | ICD-9 code: 266.2, 281.1  ICD-10 code: E53.8, D51.x  - Single occurrence on/prior to index date |
| Depression | ICD-9 code: 296.2x, 296.3x, 311;  ICD-10 code: F32.0-F32.5, F32.9, F33.0-F33.3, F33.4x, F33.9  - 2 outpatient occurrences on different days in same 12-month period or 1 inpatient occurrence on/prior to index date. |
| Any anxiety disorder | ICD-9 code: 309.81, 300.00, 300.01, 300.02, 300.23, 300.3;  ICD-10 code: F40.1x, F41.0, F41.1, F41.9, F42, F43.1x  - Composite of PTSD, panic disorder, anxiety disorder not otherwise specified, obsessive compulsive disorder, social phobia, and generalized anxiety disorder  - 2 outpatient occurrences on different days in same 12-month period or 1 inpatient occurrence on/prior to index date. |
| Alcohol abuse/dependence | ICD-9 code: 303.9x, 305.0x;  ICD-10 code: F10.x  - Single occurrence on/prior to index date |
| Any drug abuse/dependence | ICD-9 code: 304.0x, 304.1x, 304.2x, 304.3x, 304.4x, 304.5x, 304.6x, 304.7x, 304.8x, 304.9x, 305.2x, 305.3x, 305.4x, 305.5x, 305.6x, 305.7x, 305.9x  ICD-10 code: F11.x, F12.x, F13.x, F14.x, F15.x, F16.x, F18.x, F19.x  - Composite of sedative, cocaine, cannabis, amphetamine, hallucinogens, ‘other’, opioid, opioid with other SUD, other SUD excluding opioid, unspecified drug abuse/dependence.  - Single occurrence on/prior to index date |
| High health services utilization | - Controls for detection bias related to more healthcare encounters.  - Average number of outpatient clinic visits per month, calculated as total visits divided by number of months. Total visits= total number of visits in time period of interest. Number of months followed is months from first visit to last visit in time period of interest. Time period of interest is first visit in respective data time frames to index date.  - The distribution of the mean is then dichotomized into high utilizer, >75^th^ percentile vs. low utilizer, ≤75^th^ percentile. |
| Number of well visits | CPT codes: 99386, 99387, 99396 or 99397  - Number of well visits from start of data to index date. Categorized as none, 1-2, ≥3 |
| Other medications (multiple variables),  yes vs no | Anticholinergics, NSAIDs, antihypertensives, Statins, glucocorticoids, antivirals, metformin, sulfonylureas – defined by any sustained use. Sustained use is at least 2 fills in any 6 month period prior to index. |
| Age | Age in years calculated as age on index date. Categorized as 65-69, 70-74, ≥75 |
| Race (VHA only) | White, African-American, “other” (most frequently occurring in record). |
| Gender | Male, female |
| Marital status (VHA only) | Married vs. other (on/closest to index date). |
| VA only health insurance vs. VA+private/Other (VHA only) | - Insurance status variable indicating any access to non-VA health insurance. Coverage defined by most frequently occurring coverage prior to index date. MarketScan does not provide insurance coverage information. |
| Region | Home state geographic region at index. VHA provides home state, which was further categorized based on MarketScan regional categories.  Northeast (CT, ME, MA, NH, RI, VT, NJ, NY, PA)  North Central (IL, IN, MI, OH, WI, IA, KS, MN, MO, NE, ND, SD)  South (DC, DE, FL, GA, MD, NC, SC, VA, WV, AL, KY, MS, TN, AR, LA, OK, TX)  West (AZ, CO, ID, MT, NV, NM, UT, WY, AK, CA, HI, OR, WA)  Unknown |
| Year of cohort entry (retrospective cohort) | VHA: FY2011 (10/1/2010), FY2012 (10/1/2011)  MarketScan: CY2011 (1/1/2011), CY2012 (1/1/2012) |
| **Herpes Zoster Vaccine** | |
| Zoster (shingles) vaccine | Occurrence of CPT code or drug fill for vaccine product by index date.  CPT (associated drug name) codes:  90710 (Proquad) (MMRV – Measles/Mumps/Rubella/Varicella, live, subcutaneous)  90716 (Varivax) (Varicella vaccine, live, subcutaneous)  90736 (Zostavax) (Zoster vaccine, live, subcutaneous)  90750 (Shingrix) (Zoster vaccine, sub-unit, adjuvanted, intramuscular)  Drug product names: Proquad, Varivax, Zostavax, Shingrix |
| **Post-index diseases and treatments used in secondary analyses of possible mechanisms of vaccine effect on dementia outcome (these variables are not used in PS models)** | |
| Shingles disease | ICD-9 code: 053.x;  ICD-10 code: B02.x  - Single occurrence between index and end of follow-up |
| Shingles treatment | Acyclovir, Famciclovir, Valaciclovir  - Single drug claim between index and end of follow-up |

**Propensity Score (PS) and Inverse Probability of Treatment Weighting (IPTW):** A binary logistic regression model was used to calculate the PS, which is the probability of receiving a HZ vaccination given baseline covariates. Stabilized weights for each patient are computed using the PS. The stabilized weight is the marginal probability of HZ vaccination divided by the PS for HZ vaccination or (1-marginal probability of HZ vaccination) divided by (1-PS) for unvaccinated patients.^1,2^ Marginal probability is the observed probability of HZ vaccination. Using stabilized weights reduces bias associated with extreme weights (due to increased variance) and retains original sample size in analysis thereby preserving Type I error rate.^3^ Stabilized weights were trimmed if they were ≥10. Well-behaved weights have a mean close to one and a maximum less than 10 with extreme values indicating the PS model poorly specified predictors of the treatment exposure.^4,5^ We assessed balance between the HZ and no-HZ vaccination groups with standardized mean difference (SMD%). Covariates are well balanced when SMD was < 10%.^6^

**Distribution of post-HZ vaccination HZ infection and antiviral therapy:** In the VHA, 7.4% of no-HZ vaccine patients and 6.9% of patients with HZ vaccine had at least one ICD diagnostic code for HZ infection in follow-up; 5.9% of non-HZ vaccine patients and 6.9% of HZ vaccine patients had at least one fill of an antiviral treatment. In MarketScan, 4.5% of no-HZ vaccine patients and 3.4% of HZ vaccine patients had HZ infection in follow-up; 8.1% of non-HZ vaccine patients and 8.7% of HZ vaccine patients had an antiviral in follow-up.

| **Table S.2.** **Standardized mean difference percent (SMD%) comparing covariates by herpes zoster (HZ) vaccination status after inverse probability of exposure weighting, VHA and MarketScan patient data.** | | |
| --- | --- | --- |
| **Covariates, SMD%** | **VHA**  **(n=136,016)** | **MarketScan**  **(n=172,790)** |
| Index fiscal year |  |  |
| 2011 | 0.5 | 2.2 |
| 2012 | -0.5 | -2.2 |
| Age category |  |  |
| 65-69 | -0.7 | -0.1 |
| 70-74 | 0.4 | 0.4 |
| ≥ 75 | 0.4 | -0.3 |
| Female gender | 1.8 | 1.3 |
| Race |  |  |
| White | 2.2 | --- |
| Black | -2.1 | --- |
| Other | -0.8 | --- |
| Married | 2.1 | --- |
| VHA only insurance | 3.7 | --- |
| Region |  |  |
| Northeast | 0.5 | -0.1 |
| North central | -2.1 | -0.3 |
| South | 1.4 | 0.2 |
| West | 0.2 | 0.4 |
| Unknown | 0.7 | -0.7 |
| High healthcare utilization | -1.1 | 1.1 |
| # well visits, category |  |  |
| 0 | -0.3 | -1.3 |
| 1-2 | 0.3 | -0.9 |
| ≥ 3 | 0.0 | -0.6 |
| Type II Diabetes | 1.3 | -0.6 |
| Obesity | -1.0 | -0.2 |
| Hypertension | 0.4 | -0.4 |
| Stroke | 2.3 | -0.01 |
| Ischemic heart disease | 0.8 | -1.0 |
| Congestive heart failure | -0.1 | -0.5 |
| Atrial fibrillation | 3.2 | -0.1 |
| Asthma | 0.2 | 0.2 |
| COPD | 1.6 | -0.5 |
| Traumatic brain injury | 2.0 | -0.1 |
| Vitamin B12 deficiency | 2.2 | -0.7 |
| Depression | -0.6 | 0.6 |
| Anxiety disorder ^a^ | -0.1 | -0.3 |
| Nicotine dependence | -2.1 | -0.7 |
| Alcohol abuse/dependence | -1.5 | 0.1 |
| Drug abuse/dependence | -0.1 | -0.3 |
| Anticholinergics | -3.3 | 0.5 |
| NSAIDS | -3.1 | 0.2 |
| Antihypertensives | -3.7 | 0.9 |
| Statins | 0.3 | 0.7 |
| Steroids | -0.5 | -0.3 |
| Antivirals | 0.6 | 0.4 |
| Metformin | 3.2 | -0.4 |
| Sulfonylurea | -0.8 | -0.6 |

| **Table S.3. Results (HR (95%CI)) from competing risk survival models^a^ estimating the association of herpes zoster (HZ) vaccination vs. no HZ vaccination and dementia, overall and stratified by age and race** **among 136,016 VHA patients** | | | | | |
| --- | --- | --- | --- | --- | --- |
| **Group** | **Crude** | **Weighted** | **Weighted + HZ infection** | **Weighted + antiviral** | **Weighted + HZ infection + antiviral** |
|  | **HR (95% CI)** | **HR (95% CI)** | **HR (95% CI)** | **HR (95% CI)** | **HR (95% CI)** |
| Overall | 0.54  (0.52-0.56) | 0.69  (0.67-0.72) | 0.69  (0.67-0.72) | 0.69  (0.67-0.72) | 0.69  (0.67-0.72) |
|  |  |  |  |  |  |
| *Age stratified* |  |  |  |  |  |
| Age 65-69 | 0.49  (0.43-0.55) | 0.61  (0.54-0.68) | 0.61  (0.54-0.68) | 0.61  (0.54-0.68) | 0.61  (0.54-0.68) |
| Age 70-74 | 0.51  (0.46-0.56) | 0.83  (0.76-0.91) | 0.84  (0.76-0.92) | 0.83  (0.76-0.91) | 0.84  (0.76-0.92) |
| Age ≥ 75 | 0.57  (0.55-0.60) | 0.66  (0.63-0.68) | 0.66  (0.63-0.69) | 0.66  (0.63-0.68) | 0.66  (0.63-0.69) |
| *Age*HZ vaccination* ^b^ | *q=0.011* | *q=0.0002* | *q=0.0002* | *q=0.0002* | *q=0.0002* |
|  |  |  |  |  |  |
| *Race stratified* |  |  |  |  |  |
| White race | 0.55  (0.53-0.57) | 0.69  (0.67-0.72) | 0.70  (0.67-0.72) | 0.69  (0.67-0.72) | 0.70  (0.67-0.72) |
| Black race | 0.50  (0.40-0.62) | 0.70  (0.62-0.78) | 0.70  (0.62-0.78) | 0.70  (0.62-0.78) | 0.70  (0.62-0.78) |
| Other race | 0.55  (0.35-0.86) | 0.51  (0.33-0.78) | 0.51  (0.33-0.78) | 0.51  (0.33-0.78) | 0.51  (0.33-0.78) |
| *Race*HZ vaccination* ^b^ | *q=0.682* | *q=0.436* | *q=0.453* | *q=0.576* | *q=0.467* |
| HR=hazard ratio comparing HZ vaccination vs. no vaccination within each group of patients identified  ^a^ Fine and Gray (1999) method^7^  ^b^ q-value adjusted for false discovery rate | | | | | |

| **Table S.4. Results (HR (95%CI)) from Cox proportional hazards models estimating the association of herpes zoster (HZ) vaccination vs. no HZ vaccination and dementia, overall and stratified by age among 172,790 MarketScan patients** | | | | | |
| --- | --- | --- | --- | --- | --- |
| **Group** | **Crude** | **Weighted** | **Weighted + HZ infection** | **Weighted + antiviral** | **Weighted + HZ infection + antiviral** |
|  | **HR (95% CI)** | **HR (95% CI)** | **HR (95% CI)** | **HR (95% CI)** | **HR (95% CI)** |
| Overall | 0.66  (0.59-0.74) | 0.65  (0.57-0.74) | 0.65  (0.57-0.75) | 0.65  (0.57-0.74) | 0.65  (0.57-0.75) |
|  |  |  |  |  |  |
| *Age stratified* |  |  |  |  |  |
| Age 65-69 | 0.90  (0.71-1.13) | 0.95  (0.72-1.25) | 0.95  (0.72-1.25) | 0.95  (0.72-1.25) | 0.95  (0.72-1.25) |
| Age 70-74 | 0.81  (0.64-1.01) | 0.66  (0.50-0.88) | 0.67  (0.50-0.88) | 0.66  (0.50-0.88) | 0.67  (0.50-0.88) |
| Age ≥ 75 | 0.66  (0.56-0.77) | 0.56  (0.46-0.68) | 0.56  (0.87-1.31) | 0.56  (0.46-0.68) | 0.56  (0.46-0.68) |
| *Age*HZ vaccination* ^a^ | *q=0.051* | *q=0.008* | *q=0.008* | *q=0.008* | *q=0.008* |
| HR=hazard ratio comparing HZ vaccination vs. no vaccination within each group of patients identified  ^a^ q-value adjusted for false discovery rate | | | | | |

| **Table S.5. VHA and MarketScan– association between herpes zoster (HZ) vaccination and Alzheimer’s disease (AD) in follow-up, cumulative incidence % and incidence rate per 10,000 person-years (PY), patients ≥ 65 years old** | | | | | | | | | | |
| --- | --- | --- | --- | --- | --- | --- | --- | --- | --- | --- |
|  | VHA | | | | | MarketScan | | | | |
|  | Total n | AD events | | Cumulative incidence % | Incidence rate per 10,000PY | Total n | AD events | Cumulative incidence % | | Incidence rate per 10,000PY |
|  |  |  | |  |  |  |  |  | |  |
| *Overall* | *136,016* | *7,439* | | *5.5%* | *80.0/10,000PY* | *172,790* | *1,186* | *0.7%* | | *18.0/10,000PY* |
| No vaccination | 108,597 | | 6,384 | 5.9% | 88.1/10,000PY | 148,178 | 1,064 | | 0.7% | 19.4/10,000PY |
| HZ vaccination | 27,419 | | 1,055 | 3.8% | 50.4/10,000PY | 24,612 | 122 | | 0.5% | 13.0/10,000PY |
|  |  |  | | p<.001 | p<.001 |  |  | p<.001 | | p<.001 |
| Note: PY=person-years | | | | | | | | | | |

| **Table S.6. Results (HR (95%CI)) from competing risk survival models^a^ (VHA) and Cox proportional hazard models (MarketScan) estimating the association of herpes zoster (HZ) vaccination vs. no HZ vaccination and Alzheimer’s Disease** | | | | |
| --- | --- | --- | --- | --- |
|  | **Crude** | **q-value** | **Weighted** | **q-value** |
| VHA | 0.59 (0.56-0.63) | 0.0002 | 0.75 (0.71-0.80) | 0.0002 |
|  |  |  |  |  |
| MarketScan | 0.63 (0.52-0.76) | 0.0002 | 0.70 (0.55-0.88) | 0.004 |
|  |  |  |  |  |
| ^a^ Fine and Gray (1999) method^7^  ^b^ q-value adjusted for false discovery rate | | | | |

Figure 1a. Veterans Health Administration eligibility criteria

Non-HZ vaccine group remains free of HZ vaccine in follow-up

(No vaccine – n=99,708)

(HZ vaccine – n=19,242)

Index Date=10/1/2010 (FY2011)

≥3 well visits FY09-FY19, age ≥50 at first well visit (n=458,460)

HZ vaccine status at 10/1/2010:

(No vaccine – n=432,133)

(HZ vaccine – n=26,327)

≥65 years old at index

(No vaccine – n=251,027)

(HZ vaccine – n=21,562)

Enrollment 2 years prior to index

(No vaccine – n=251,027)

(HZ vaccine – n=21,562)

Free of dementia and medications for 2 years prior to index

(No vaccine – n=175,004)

(HZ vaccine – n=19,479)

> 90 days follow-up

(No vaccine – n=165,934)

(HZ vaccine – n=19,242)

Non-HZ vaccine group remains free of HZ vaccine in follow-up

(No vaccine – n=11,414)

(HZ vaccine – n=8,280)

Index Date=10/1/2011 (FY2012)

Sample that is not eligible 10/1/2010 (n=339,510)

HZ vaccine status at 10/1/2011:

(No vaccine – n=322,106)

(HZ vaccine – n=17,404)

≥65 years old at index

(No vaccine – n=161,280)

(HZ vaccine – n=11,094)

Enrollment 2 years prior to index

(No vaccine – n=109,336)

(HZ vaccine – n=10,751)

Free of dementia and medications for 2 years prior to index

(No vaccine – n=85,227)

(HZ vaccine – n=8,514)

> 90 days follow-up

(No vaccine – n=79,683)

(HZ vaccine – n=8,280)

Total (n=138,644)

Remove missing demographics

(n=136,016)

Footnote Figure 1a. Patients not eligible in FY2011 were used to sample for FY2012 index date

Figure 1b. MarketScan eligibility criteria

Non-HZ vaccine group remains free of HZ vaccine in follow-up

(No vaccine – n=103,073)

(HZ vaccine – n=12,895)

Index Date=1/1/2011 (CY2011)

≥3 well visits CY09-CY19, age ≥50 at first well visit (n=4,989,703)

HZ vaccine status at 1/1/2011:

(No vaccine – n=4,923,533)

(HZ vaccine – n=66,170)

≥65 years old at index

(No vaccine – n=180,746)

(HZ vaccine – n=13,837)

Enrollment 2 years prior to index

(No vaccine – n=138,538)

(HZ vaccine – n=13,833)

Free of dementia and medications for 2 years prior to index

(No vaccine – n=133,828)

(HZ vaccine – n=13,390)

> 90 days follow-up

(No vaccine – n=130,396)

(HZ vaccine – n=12,895)

Non-HZ vaccine group remains free of HZ vaccine in follow-up

(No vaccine – n=45,105)

(HZ vaccine – n=11,717)

Index Date=1/1/2012 (CY2012)

Sample that is not eligible 1/1/2011 (n=4,873,735)

HZ vaccine status at 1/1/2012:

(No vaccine – n=4,747,412)

(HZ vaccine – n=126,323)

≥65 years old at index

(No vaccine – n=128,202)

(HZ vaccine – n=16,721)

Enrollment 2 years prior to index

(No vaccine – n=106,951)

(HZ vaccine – n=16,712)

Free of dementia and medications for 2 years prior to index

(No vaccine – n=101,435)

(HZ vaccine – n=15,947)

> 90 days follow-up

(No vaccine – n=80,595)

(HZ vaccine – n=11,717)

Total (n=172,790)

Footnote Figure 1b. Patients not eligible in CY2011 were used to sample for CY2012 index date

**REFERENCES**

1. Rosenbaum PR, Rubin DB. The central role of the propensity score in observational studies for causal effects. Biometrika 1983;70:41-55.

2. Curtis LH, Hammill BG, Eisenstein EL, Kramer JM, Anstrom KJ. Using inverse probability-weighted estimators in comparative effectiveness analyses with observational databases. Med Care 2007;45:S103-7.

3. Xu S, Ross C, Raebel MA, Shetterly S, Blanchette C, Smith D. Use of stabilized inverse propensity scores as weights to directly estimate relative risk and its confidence intervals. Value Health 2010;13:273-7.

4. Harder VS, Stuart EA, Anthony JC. Propensity score techniques and the assessment of measured covariate balance to test causal associations in psychological research. Psychol Methods 2010;15:234-49.

5. Stürmer T, Wyss R, Glynn RJ, Brookhart MA. Propensity scores for confounder adjustment when assessing the effects of medical interventions using nonexperimental study designs. J Intern Med 2014;275:570-80.

6. Austin PC, Stuart EA. Moving towards best practice when using inverse probability of treatment weighting (IPTW) using the propensity score to estimate causal treatment effects in observational studies. Stat Med 2015;34:3661-79.

7. Fine JP, Gray RJ. A Proportional Hazards Model for the Subdistribution of a Competing Risk. Journal of the American Statistical Association 1999;94:496-509.
