# Supplementary material for: Impact of herpes zoster vaccination on incident dementia: A retrospective study in two patient cohorts
Source: PLoS One. 2021 Nov 17;16(11):e0257405. doi: 10.1371/journal.pone.0257405 (PMC8597989; doi:10.1371/journal.pone.0257405)
Supplement: Attachment — Submitted filename: response to critiques PLOS ONE final.docx [file pone.0257405.s002.docx]

RESPONSE TO CRITIQUES

**Editor comments**

**1) Format manuscript to PLOS ONE style**

We have formatted the manuscript to adhere to PLOS ONE style

**2.Please provide additional details regarding participant consent. In the ethics statement in the Methods and online submission information, please ensure that you have specified (1) whether consent was informed and (2) what type you obtained (for instance, written or verbal, and if verbal, how it was documented and witnessed). If your study included minors, state whether you obtained consent from parents or guardians. If the need for consent was waived by the ethics committee, please include this information. If you are reporting a retrospective study of medical records or archived samples, please ensure that you have discussed whether all data were fully anonymized before you accessed them and/or whether the IRB or ethics committee waived the requirement for informed consent. If patients provided informed written consent to have data from their medical records used in research, please include this information.**

We have added the following statement to the Methods and Ethics Statement: The Saint Louis University IRB approved the research as a non-human subjects study because this was a retrospective cohort study of anonymized medical record and claims data. Investigators only had access to anonymized data.

**Once you have amended this/these statement(s) in the Methods section of the manuscript, please add the same text to the “Ethics Statement” field of the submission form (via “Edit Submission”).**

We report this statement in the submission form.

**3. Please modify the title to ensure that it is meeting PLOS’ guidelines (https://journals.plos.org/plosone/s/submission-guidelines#loc-title). In particular, the title should be "specific, descriptive, concise, and comprehensible to readers outside the field" and in this case we feel it is not informative and specific about your study's scope and methodology.**

We changed the title to: “Impact of Herpes Zoster Vaccination on Incident Dementia: A Retrospective Study in Two Patient Cohorts”

**4.We note that you have indicated that data from this study are available upon request. PLOS only allows data to be available upon request if there are legal or ethical restrictions on sharing data publicly. For information on unacceptable data access restrictions, please see http://journals.plos.org/plosone/s/data-availability#loc-unacceptable-data-access-restrictions**.

We now submit a Data Availability Statement: Veterans Health Administration (VHA) may not be shared without interested persons obtaining VHA IRB and Data Access Request approvals. MarketScan data is proprietary and the authors are prevented from sharing data per data use agreement.

**5.Thank you for stating the following in the Acknowledgments Section of your manuscript:**

**"Support for VA/CMS data is provided by the Department of Veterans Affairs, Veterans Health**

**362 Administration, Office of Research and Development, Health Services Research and**

**363 Development, VA Information Resource Center (Project Numbers SDR02-237 and 98-004). This**

**364 material is the result of work supported with resources and the use of facilities at the Harry S.**

**365 Truman Memorial Veterans' Hospital**

**366 The funder had no role in the design and conduct of the study; collection, management, analysis,**

**367 and interpretation of the data; preparation, review, or approval of the manuscript; and decision to**

**368 submit the manuscript for publication."**

**We note that you have provided funding information that is not currently declared in your Funding Statement. However, funding information should not appear in the Acknowledgments section or other areas of your manuscript. We will only publish funding information present in the Funding Statement section of the online submission form.**

**Please remove any funding-related text from the manuscript and let us know how you would like to update your Funding Statement. Currently, your Funding Statement reads as follows:**

**"Benter Foundation award 2020-01 to JFS**

**https://benterfoundation.org/**

**The sponsor played no role in study design, data collection, analysis, decision to publish or preparation of the manuscript"**

**Please include your amended statements within your cover letter; we will change the online submission form on your behalf.**

We removed the funding statement from the Acknowledgements and we do not need to revise the funding statement in the online submission.

**6. Please include captions for your Supporting Information files at the end of your manuscript, and update any in-text citations to match accordingly. Please see our Supporting Information guidelines for more information: http://journals.plos.org/plosone/s/supporting-information.**

A list of captions for supporting information is now presented at the end of our manuscript.

**7. Additional Editor Comments:**

**Overall, this study is a well-designed study to evaluate the association between herpes zoster vaccination and dementia. The relationship between herpes zoster vaccination and dementia type (such as Alzheimer's disease versus non-Alzheimer's disease or vascular dementia) will be interesting. Some discussion can surround this issue.**

We expanded the Discussion, paragraph 1, with the following statements: Our sensitivity analysis indicate that the reduced risk for dementia also applies to AD. While the magnitude of effect was marginally less for risk of AD, our results support an association between HZ vaccination and reduced risk for dementia, including AD. Vaccinations may reduce risk for other forms of dementia, such as vascular dementia, by lowering inflammation which is common in cardiovascular conditions.

**Reviewer #1: I have read the manuscript Herpes Zoster Vaccination and Dementia. Overall, this is an interesting and well performed study with a timely and important research question. Major strengths is the large sample size, use of two different cohorts and long follow-up time. The statistical analysis is sound. Major limitations are discussed and the conclusions are fair. I recommend this manuscript is sent for revision.**

Thank you for the kind remarks.

**1) Previous studies on vaccinations and dementia all has major problems and should be discussed taking this into account. That having problems remembering things is associated with dementia development is not surprising, and therefore to rely on self-reported vaccinations is not without problems. Some other studies have very short follow-up time. As dementia disorders develop over several years before diagnosis, an effect seen in only one year for sure do not represent an actual causal effect but some confounding or reverese causality effect.**

We mention the limitations of existing research in the Introduction and now add additional comments about these studies in the Discussion, lines 326-328.

**When referring to studies on infections and dementia, herpes simplex type one should be mentionned. Compared to Pertussis, there is very much more epidemiological support for a link to herpes simplex, see for example cohort studies by Letenneur 2008, Lovheim 2015, Lopatko-Lindman 2019, Linard 2020, and Itzhaki 2016 "Microbes and Alzheimer's Disease" for a review. I do not think Bordetella Pertussis deserves to be mentioned at all, it is only one of many pathogens suggested to be linked to AD development (other examples are chlamydia pneumonie, borrelia, toxoplasma, picorna virus and many others). Only HSV1 has been shown to associate with increased risk in several different cohort studies. VZV could also be linked to vascular diseases and stroke. Maybe could this contribute to an effect on vascular dementa, and should possibly be discussed also?**

We revised this section of the Introduction which now reads: “Numerous bacterial and viral infections have been associated with incident dementia and or AD[2, 3]. Among these, the largest body of evidence supports a link between herpes zoster (HZ) infection and increased risk for dementia[5-10].”

Additional references suggested by the reviewer have been added.

**Reviewer #2:** **The research design and analysis approach author took is very suitable, but there are some relatively some minor issuess that needs attention, therefore provided advice as follow:**

**(1)The title of the research is too simple, it is recommended to provide more information, for example the research design.**

See response to Editor’s comment

**(2)Typesetting of the 282 line.**

I could not find a typesetting issue in line 282. But I did add an indent to line 283.

**(3)The p-value location in Table 2 is strange, needs adjustment after verifying with the author.**

There was one p-value that we moved into the correct row. Otherwise we are not sure what the reviewer saw in Table 2.

**(4)Please add hypothesis testing p-value of comparison incidence rate in Table 3**

**and Table S.5.**

We have now added p-values for cumulative incidence and incidence rate in Tables 3 and S.5.

**(5)Supplementary Table 3 and Table 4 Lacks the description for method of how to choose comparison group to do modeling; lacks the report of HZ infection original number of people, unsure of the result if HZ infection is weighted.**

We have tried to add more description to these tables (adding rows to describe models – age stratified vs. race stratified, adding HR column headers). We have added a footnote indicating that the hazard ratio within each group of patients identified is comparing HZ vaccination vs. no vaccination. The results in these tables include crude and various weighted models as indicated in the column headers. These tables present information included in the forest plot Figure 1.

**(6)There might have been a typographical error on supplementary Figure1b CY09~FY19 , needs adjustment after verifying with the author.**

Thank you for catching this error. We have corrected it.

**(7)Supplementary Figure1a Figure1b both specially mentioned that samples are divided into two groups of eligible or not eligible, but why not fails to further analysis?**

The sampling scheme used 2 index dates to identify patients, either CY2011 (FY2011 in VHA) or CY2012 (FY2012 in VHA). Patients that were not eligible on the first index date (as indicated by the boxes “Sample that is not eligible 10/1/2010” (VHA) or “Sample that is not eligible 1/1/2011” (MarketScan)) were assessed for eligibility at the next index date. Also for clarity, on page 7 lines 119-120 of the manuscript, we have mentioned that “Patients not meeting eligibility criteria in 2011 were used to sample for the 2012 index date”. We have added footnotes to both figures with similar verbiage to add clarity to these figures. This sampling scheme tries to show that a patient entered the cohort the first time he or she met eligibility criteria (either at the first or second index date).
